# Navigating the Algorithm: A Narrative Review of Social Media’s Impact on Mental Health in Clinical and Non-Clinical Adolescent Populations

**DOI:** 10.3390/children13070872

**Published:** 2026-06-30

**Authors:** Andreea Socol, Lucia Emanuela Andrei, Catrinel Maria Dijmarescu, Diana Dragomir, Alexandra-Diana Iotu, Ilinca Mihailescu, Florina Rad

**Affiliations:** 1 Child and Adolescent Psychiatry Department, “Carol Davila” University of Medicine and Pharmacy, 020021 Bucharest, Romania; andreea.gherghe@drd.umfcd.ro (A.S.); maria-catrinel.dijmarescu0925@rez.umfcd.ro (C.M.D.); diana.dragomir@rez.umfcd.ro (D.D.); ilinca.mihailescu@umfcd.ro (I.M.); florina.rad@umfcd.ro (F.R.); 2 Child and Adolescent Psychiatry Department, “Prof. Dr. Alexandru Obregia” Clinical Psychiatry Hospital, 041914 Bucharest, Romania

**Keywords:** child and adolescent psychiatry, social media, adolescents, mental health, digital affordances, internalizing disorders, externalizing disorders

## Abstract

Background: In recent years, there has been a growing concern regarding social media driving the decline of mental health, especially among adolescents. However, scientific consensus remains mixed, with many studies reporting only small or inconsistent associations. Aims: This paper aims to present the latest and most influential findings in the field of social media, with a focus on understanding the impact it has on adolescents’ mental health by looking at clinical versus non-clinical populations. Method: We conducted a comprehensive search through Scopus, looking for scientific articles and reviews published from January 2020 to March 2026 that include social media and adolescents with mental health conditions. We examined social media use patterns, affordances, mechanisms of impact, and clinical versus non-clinical populations. Results: There is limited literature comparing clinical versus non-clinical adolescent populations. Adolescents with mental health disorders spend more time online, teens with internalizing conditions report being more prone to social comparison and more sensitive to digital feedback, while those with externalizing conditions report a lack of control over how much time they spend on social media. Screen time alone is not sufficient to determine the impact on mental health. Among the features that might be associated with mental health problems are sharing personal content and scrolling through others’ posts. Conclusions: The impact of social media could be shaped by pre-existing vulnerabilities. There is a need for longitudinal study designs to test temporal associations and more research to cover the gap on clinical populations to develop better policies and interventions.

## 1. Introduction

Rates of depression and anxiety have risen among adolescent populations in the last 20 years, especially in high-income countries [1,2], coinciding with the pervasive integration of social media platforms into their daily lives. According to the Global Burden of Disease Study from 2021, 1 in 7 adolescents (15.2%) suffers from at least one mental disorder, the most prevalent being anxiety [3]. Between 2019 and 2021, 69.5% of adolescents aged 11 to 15 reported having at least one social media account, the most popular platforms among them being TikTok, YouTube, and Instagram [4]. The legal minimum age required for opening a social media account in most countries is 13 [5]. Nevertheless, research has found that, in the US, children under 13 had an average of 3.38 social media accounts [4].

Most teenagers today navigate the algorithms of social media daily. The average daily time 13-to-18-year-olds spend on social media has risen to 1 h and 27 min from 1 h and 10 min four years ago [6]. Preadolescents (8–12 years old) report using screens for entertainment for around 5.5 h daily, while teenagers (13–18 years old) spend around 8.5 h daily doing various digital activities [6]. Adolescent girls exhibit a higher usage of TikTok, Snapchat, Instagram, and Pinterest (platforms that are centered on curated self-presentation and social comparison), while boys report higher usage of YouTube and Reddit (where there is a lower emphasis on looks and a higher degree of anonymity) [4].

Young people engage with social media more than adults [7], the exposure being higher for older adolescents than younger adolescents [8], and research suggests that they may be more vulnerable to the harmful impact of social media on their mental health in comparison to older age groups [9].

Considering the above, in recent years there has been a widespread growing concern regarding social media driving the decline of adolescents’ mental health. The temporal association between the two has spurred extensive research trying to understand whether there is a correlation linking social media use to the rise in mental health conditions in adolescents. A recent meta-analysis published in 2026 by Teague et al. synthesizing 153 longitudinal studies identified a modest but consistent association between social media use and depression, self-harm ideation, externalizing and internalizing behaviors, drug use, problematic internet use, and poor developmental outcomes [10]. Some countries have already started enacting restrictions such as prohibiting adolescents under 16 from creating social media profiles, requiring parental consent for access, and mandatory age verification [11]. However, scientific consensus remains mixed, with many studies reporting only small or inconsistent associations [12].

Despite these findings, clinical work suggests that adolescents with pre-existing mental health conditions might be affected differently [13]. Nevertheless, scientists have largely overlooked these adolescents until recently. Lately, research is shifting towards comparing patterns of use in clinical versus non-clinical populations [13] and focusing on the way affordances are linked to mental health vulnerabilities [14]. Additionally, in recent years the algorithms of social media platforms and the way users interact with them have advanced so rapidly that studies published more than five years ago fail to include the applications that are currently most popular, such as TikTok. Therefore, this narrative review aims to explore the latest and most influential findings in the field of social media, with a focus on understanding the impact it has on adolescents’ mental health by looking at clinical versus non-clinical populations. 

## 2. Methods

We conducted a comprehensive search through Scopus looking for scientific articles and reviews published from January 2020 to March 2026 that include social media’s impact on adolescents with mental health conditions. The following keywords, filters, and Boolean operators were used for the search: TITLE-ABS-KEY ((social media OR TikTok OR Instagram OR Snapchat OR Facebook) AND (impact) AND (“mental health” OR depress* OR anxie* OR “eating disorder*” OR “self-harm”) AND (clinical OR patient* OR diagnos* OR “non-clinical” OR “control group”) AND (adolescen* OR teen* OR youth)) AND PUBYEAR > 2019 AND PUBYEAR < 2027 AND NOT (COVID) AND (LIMIT-TO (LANGUAGE, “English”)). To prioritize the assessment of long-term trends in social media usage and its effects on adolescent mental health, literature specific to the COVID-19 pandemic was excluded, as such data often reflects the unique and acute psychological stresses of the global crisis rather than typical patterns of engagement.

**Figure 1:**
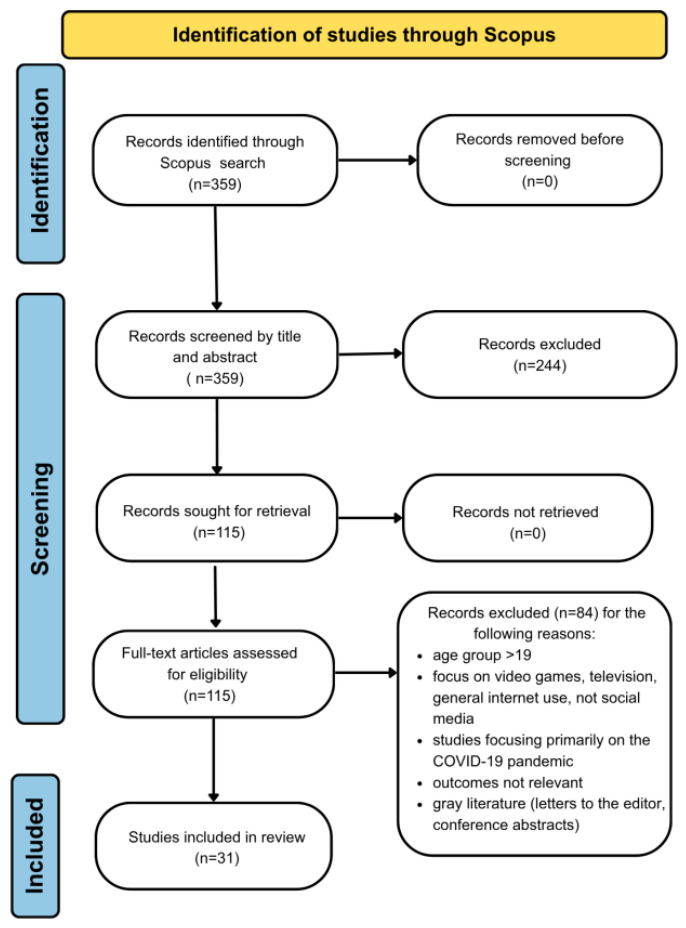
Flow chart of data collection.

Initially, 359 titles and abstracts were screened from which 115 full-text papers were retrieved and further analyzed for eligibility. Peer-reviewed original articles, narrative and systematic reviews published in English were included to provide a comprehensive synthesis of the available literature. Additional criteria for inclusion were: (i) studies involving children aged up to 19 years, (ii) studies addressing the impact of social media on adolescent populations (clinical, community samples, non-clinical, clinical vs. non-clinical), and (iii) papers presenting novel research approaches on the topic of social media’s impact on children (patterns of use, affordances). In order to prioritize research reflecting the current digital landscape, studies were considered ineligible if they were: (i) published before 2020, (ii) conducted on adult populations, (iii) gray literature, and (iv) focused exclusively on the COVID-19 pandemic. After reading the full text, 31 articles met our inclusion criteria and were incorporated in the review (Figure 1). In addition to the Scopus search, we manually screened the reference lists of eligible articles to identify potentially relevant papers to ensure an in-depth analysis of the subject. These studies were assessed using the same eligibility criteria as those identified through the database search. One relevant study published in 2017 was retained despite falling outside the predefined publication period because of its relevance to the review topic.

Findings have been structured thematically, focusing on social media use patterns, affordances, and their impact on clinical versus non-clinical populations. 

## 3. Results

Due to a paucity of research on clinical samples in social media [13], the findings presented in Section 3.1, Section 3.2 and Section 3.3 are derived primarily from studies conducted in general adolescent populations. Evidence from clinical populations is presented in Section 3.4.

### 3.1. Social Media Use Patterns in Adolescence

This section explores specific types of engagement (active vs. passive), gender-based content preferences and their impact, and how different algorithms and affordances shape behavior differently.

#### 3.1.1. Beyond “Screen Time”

A fundamental topic in the current literature is the distinction between active versus passive online engagement. Active engagement such as chatting and messaging is hypothesized to build social networks and strengthen personal connections, thereby possibly acting as a resilience factor for adolescent well-being. On the contrary, passive consumption, such as endless scrolling and silent monitoring of feeds, can often trigger social comparison and envy [15,16]. However, evidence remains highly dependent on the impact of individual differences and baseline vulnerabilities [16,17].

Through a longitudinal study, Tibbs et al. reveal a paradox in the “frequency vs. function dynamic”: while high-frequency interactive users are associated with a higher psychological risk, temporary peaks of active communication can significantly help reduce internalizing symptoms, suggesting that active engagement can be a short-term emotional regulator despite the persistent complexity of long-term data. Notably, while passive online engagement is associated with poorer mental health, Tibbs et al. found no longitudinal data that supports the exacerbation of internalizing difficulties over time, highlighting that the narrative of “passive is always harmful” may be an oversimplification [18].

In line with this transition towards a more precise analytical framework, Munzer et al. argue that durations of social media use have become an inadequate proxy for adolescent well-being, highlighting that even passive consumption is far from a homogenous experience. For instance, nostalgic reminiscing, such as viewing photographs of shared experiences with close friends, can actually enhance well-being. Furthermore, data suggests that sophisticated algorithmic curation and the user’s psychological intent act as better predictors of well-being than mere patterns of use [19].

#### 3.1.2. Gender Dynamics and Content Trends

The adolescent digital landscape is highly fragmented, manifesting in both platform preference and behavioral patterns. Empirical data highlights a significant divide in gender dynamics and their patterns of use. Female adolescents appear to have a marked predisposition toward visually driven and interactive platforms such as TikTok (77.7%), Instagram (69.6%), and Snapchat (66.5%). In contrast, male peers demonstrate a clear affinity for YouTube (71.7%), suggesting that gender influences the performative (expressing themselves actively) vs. consumptive (consuming content passively) use of social media [4,20].

However, research by Manago highlights that these patterns are not primarily a product of biological sex, but are rather mediated by internalized gender beliefs. For instance, feminine ideology predicts the use of social media for emotional connection and appearance validation, while masculine ideology is a primary driver of “competitive activity bonding”. Both sets of beliefs contribute to social compensation, where digital spaces act as a strategic platform to navigate and overcome social challenges [21].

The negative effects of these trends are shaped by gender identity, which is a strong moderator of mental health outcomes. A longitudinal analysis of over 58,000 adolescents shows that the impact is most pronounced among cisgender girls. For transgender and gender-diverse youth the relationship is dual: social media can facilitate identity affirmation while simultaneously exposing them to psychosocial risks. These findings suggest that understanding digital risk requires an analysis of how individual identity and algorithm curation interact with each other [22].

#### 3.1.3. Platform Specificity

Recent research suggests that the impact of social media is not homogenous but rather shaped by platform specificity, underscoring the unique characteristics that mediate how the user interacts with the platform. According to the authors, it is essential to shift from the analysis of cumulative “screen time” to specific algorithmic affordances, as various platforms shape different emotional experiences. Older platforms like Facebook were mainly engineered to facilitate direct communication and maintain close connections, while modern platforms such as TikTok and YouTube use predictive algorithms optimized for sustained passive consumption [20].

TikTok represents a transition from traditional networking to a fast-paced, short-duration video sequence that creates a distinct type of digital consumption and engagement [23,24]. These findings suggest that engaging tools such as an “endless scroll” create a captivating experience that makes it difficult for teenagers to put down their phones [24]. Additionally, when it comes to vulnerable adolescents at risk, TikTok’s algorithm seems to pick videos and act as an “echo chamber”; for example, an adolescent interested in diets and fitness will keep being exposed to more related content [14,23,24,25]. Furthermore, the visually driven affordances of TikTok and Instagram seem to fundamentally redefine cognitive processes related to upward social comparison, facilitating a baseline for negative self-evaluation. Research suggests that this algorithmic architecture is highly detrimental to adolescents with a heightened susceptibility to negative emotionality, as it exacerbates negative self-evaluation through constant exposure to unrealistic standards [20]. 

### 3.2. Digital Affordances

The concept of digital affordances in understanding adolescent social media use is relatively recent and increasingly more present in current research. Affordances, defined as the action possibilities and design features of digital technology, interact with adolescents’ development in a period defined by neurobiological vulnerability, an increased socio-affective reactivity associated with an immature regulatory control [14]. Table 1 presents the findings of a review that reported 13 main affordances of social media platforms and the underlying behavioral, cognitive, and neurobiological mechanisms by which each one amplifies adolescents’ mental health vulnerabilities [14].

Subsequently, when integrating the impact of multiple digital affordances, key characteristics like quantifiability, visibility, and persistence emerge. Table 2 synthesizes data on three affordances and their proposed relevance to adolescent mental health. Much of the current literature is based on general adolescent samples; therefore, the proposed relevance for mental health should be interpreted according to the study populations represented, as direct evidence from clinically diagnosed adolescents remains limited.

### 3.3. Neurobiological Mechanisms

High neuroplasticity during adolescence, especially regarding social and reward pathways, may increase adolescents’ sensitivity to social media rewards, such as likes or followers [14,30].

A three-year longitudinal study using functional magnetic resonance imaging suggests that habitual checking of social media may modify neural sensitivity of the social reward pathway, with quantifiable alterations in the amygdala, insula, ventral striatum, and dorso-lateral prefrontal cortex during social feedback anticipation [31]. Conversely, users that do not check habitually appear to present a normative process of decreasing sensitivity to social anticipation once neural maturity is reached [31]. However, these hypotheses are not supported by other studies investigating brain development and screen use as they report no impact on functional brain organization [14].

Several studies revealed that problematic, excessive use of digital platforms may be associated with brain alterations, such as decreased gray matter in nucleus accumbens, amygdala, and insula, potentially resembling changes similar to those observed in addictions. Moreover, research suggests that inhibitory regulation may also be impaired [32,33].

Nevertheless, evidence linking specific patterns of social media use to structural neuroimaging markers remains limited, and causal pathways have yet to be established [14]. Future longitudinal neuroimaging studies are needed to clarify how social media affordances interact with neurodevelopmental processes during adolescence.

### 3.4. Clinical Versus Non-Clinical Populations

Research on clinical versus non-clinical adolescent populations remains limited [34]. Most frequently, data derive from community samples where adolescents are divided into two categories, high-symptom and low symptom groups, without records of diagnosed psychiatric disorders or functional impairment. According to a systematic review by Fassi, only 11% of the studies examined adolescents with diagnosed mental health disorders [34]. A positive and significant association was found between social media and internalizing symptoms in those samples. However, similar associations were found in non-clinical samples [34]. A more recent study by the same author that consisted of adolescents who were clinically assessed for mental health disorders reported that teenagers with mental health conditions tended to spend more time on social media platforms compared to their peers with no conditions [13]. Additionally, they reported reduced satisfaction with the number of online friends [13]. When it comes to adolescents with clinical internalizing conditions (anxiety, depression, eating disorders) versus adolescent populations with no clinical conditions, the clinical group was found to report higher social comparison (comparing themselves to others online, engaging in upward comparison, evaluating their appearance, popularity, lifestyle, and achievements against peers), a greater affective impact from feedback (likes, comments, views, and reactions were associated with a greater influence on their emotions), and were less open and truthful about self-disclosure (more likely to conceal aspects about themselves, less willing to share their true thoughts, more cautious and selective) [13]. Among adolescents with mental health disorders, those with internalizing disorders differed from those with externalizing conditions by engaging more in online social comparison, spending more time on social media, and reporting greater dissatisfaction with their number of online friends, while most other aspects of social media use appeared similar between the two samples [13]. However, data directly comparing these sub-groups relies heavily on a limited number of studies and should be interpreted with caution. Nonetheless, these findings suggest that key variations in social media use among adolescents with mental health conditions may be related to the way online interactions are perceived and integrated into their emotional well-being, representing an important avenue for future research.

Throughout the following sections, we distinguish between findings derived from clinically diagnosed samples and community samples. This distinction is important because the clinical significance and the ability to generalize the findings may vary across these populations.

#### 3.4.1. Anxiety and Depression

In a longitudinal study involving 2350 adolescents from the general population, the findings indicate that more than 3 h per day of social media use was associated with increased levels of depressive and anxiety symptoms over time and a higher likelihood of clinically significant manifestations. Reduced sleep duration and prolonged sleep onset latency were found to partially mediate these relationships [35]. However, this might be a bidirectional association with insufficient sleep leading to a higher use of social media.

Depressive symptoms and a sense of loneliness seem to be associated with a more intense passive usage of social media in an attempt to alleviate low mood that can trap adolescents in a cycle that maintains or worsens those negative feelings [30]. Among adolescents receiving care for depression, suicidal ideation, or suicidal behavior it was found that 40.3% of them reported problematic social media use, which was associated with more severe mental health symptoms as well as lower resilience, poor social adjustment, and poor overall functioning [36]. This is consistent with the findings of a study published in 2025 conducted on outpatients from a mental health clinic which showed that young people who spend more time online tend to have higher levels of anxiety and loneliness compared to other age groups [37].

#### 3.4.2. Self-Harm Thoughts and Behaviors

Regulating emotions when feeling distressed is one of the main reasons adolescents engage in self-harm [38]. Research shows that teenagers from community samples who experience cybervictimization (being bullied or harassed online) are exposed to or engage with self-harm-related content more often, and are more likely to have self-injurious thoughts and behaviors [39]. A study that compared adolescents with a history of non-suicidal self-injury behavior to a healthy control group found that, among adolescents who engage in self-harm, negative events on social media showed a stronger positive association with the way they perceive stress, negative emotions, and the urge to harm themselves compared to stressful real-life events. This might suggest that, for high-risk adolescents, online experiences could have a more potent impact on their mood and thoughts of self-harm than stressors offline [40]. Out of a group of patients hospitalized with suicidal ideation or for a recent suicide attempt, 40% of them reported difficulties controlling social media use and a feeling of being “addicted”, while 37% of them mentioned experiencing pressure to maintain metrics on various platforms (likes, posts, followers) [13,41]. However, these findings from hospitalized or high-risk patients may not be representative of all adolescents with self-injurious thoughts and behaviors, particularly those identified in community or outpatient settings.

#### 3.4.3. Eating Disorders

The majority of eating disorders occur during adolescence, with this developmental stage being characterized by numerous body changes, increased body awareness, high susceptibility to social comparison, peer influence, and the self-esteem developments. A total of 80% of adolescent girls from a community population study report that social media use, especially Instagram and TikTok, has a negative impact on how they feel about their body [42]. Blanchard concludes in a systematic review that there is a significant association between social media and general disordered eating symptoms in community samples [43]. Longitudinal evidence gathered from adolescents from the general population suggests that social media use predicts increases in eating-disorder-related symptoms over time, this association being partially mediated by a decrease in self-esteem [44]. However, neither study differentiates between clinical versus non-clinical samples nor can support causality.

Research on clinical patients with anorexia nervosa shows that adolescent girls with this eating disorder spend more than 3 h on social media every day [45], more than the average of their healthy peers [6]. Those who spend five or more hours on social media a day seem to have limited connections with peers online, more severe clinical symptoms, and a more altered perception of their body [45]. Instagram is one of the most popular platforms among them [45], a platform well known for promoting perfectly tailored body figures, setting up unrealistic beauty standards, and thus fostering a comparison culture [42,46]. Girls diagnosed with anorexia nervosa report being exposed online to selective content such as pro-anorexia posts that encourage restrictive diets, content encouraging physical activity, and content that portrays a specific slender physique, all of these being associated with an excessive preoccupation with their eating habits and appearance [47].

#### 3.4.4. Externalizing Conditions

These pathologies have been studied less extensively in relation to social media use in comparison to internalizing disorders. Adolescents with externalizing disorders such as ADHD or conduct disorder showed an increase in how much time they spend on social media platforms when compared to healthy teenagers [13]. Patients with ADHD seem to also use the internet in a more problematic manner (excessive gaming, spending more time on social media, etc.) compared to healthy controls without ADHD [48,49]. This pattern of use is bidirectionally associated with more severe symptoms of ADHD, which might be explained by the shared neurological impulse control pathways between addictive behaviors and this neurodevelopmental disorder [50].

### 3.5. Not All Social Media Use Is Harmful

Although social media is frequently linked to harmful effects, there are numerous positive aspects of it that should not be overlooked, especially when it comes to how they are used and the quality of the interactions [51,52,53]. Results from a qualitative study show that 67% of patients hospitalized for suicidal behavior say social networks help adolescents keep in touch with friends and family, 60% of them value that it offers access to positive content (such as funny or cute animal videos or other relatable memes), and 53% of them use it in order to access social support. Often teens with internalizing conditions have difficulties making and maintain friendships offline, therefore social media applications offer a space for seeking support from online networks and reducing isolation [41,53]. Similarly, teens from rural areas, at risk of isolation, or teens from vulnerable minorities such as the LGBT+ community seem to be the groups benefitting the most from online social support [54]. Additionally, social media seems to be an important source of information about mental health for teenagers [55].

## 4. Discussion and Conclusions

This narrative review highlights the complexity of the relationship between social media and mental health and underscores its dependence on individual vulnerabilities, patterns of engagement, and specific affordances of various platforms.

In order to accurately assess the impact of the digital world on adolescents’ mental health, we must look beyond the single paradigm of screen time. Adolescents do not interact with social media in a universal manner; rather, social media is a heterogeneous ecosystem where adolescents can actively participate (e.g., direct messaging, content creation) or passively consume content (e.g., endless scrolling). Furthermore, AI-driven algorithms that curate personalized content based on one’s interests have reshaped how youth navigate social media and what they are exposed to. Content related to mental disorders and users’ experience with these conditions has grown in popularity in recent years, and might contribute to the social contagion of symptoms among vulnerable youth [23]. While the earlier research focused on the number of hours spent online, newer evidence suggests that duration alone is a poor instrument for psychological outcomes [16]. Additionally, when compared to other psychological stressors such as bullying, family support deficits, and schoolwork dissatisfaction, time spent on social media tends to show a weaker association [56].

A nuanced perspective is required—one that focuses on the nature of engagement and the architectural affordances of different platforms. Therefore, a better understanding of adolescent digital life requires focusing on specific behaviors and experiences such as type of use, content, context, and motivation rather than treating all use as a single, undifferentiated activity [19,57].

Overall, the available evidence suggests that, in some studies, adolescents with mental health conditions seem to differ from healthy adolescents, not only by spending more time navigating social networks but especially in how strongly they react to social media platforms, especially around feedback, comparison, and problematic engagement [13]. Across several diagnostic groups, adolescents with mental health conditions report higher levels of online social comparison, increased emotional reactivity to digital interactions and feedback, and greater engagement with disorder-specific content [45,47,48,49]. However, these differences are not consistent across all conditions and much of the existing evidence is derived from community samples rather than populations with formal clinical diagnoses.

Authentic clinical samples consisting of adolescents with mental health disorders recruited from psychiatric clinics are rare in social media research [34]. When data comes primarily from community samples and influences public health policies, it might underestimate the risks and overlook the needs of the most vulnerable populations.

Although data remains limited and most studies conducted on clinical populations have small participant samples, they highlight that there are individual differences in susceptibility to negative social media effects, and that one of the factors that could influence it might be pre-existing mental health conditions [13,39,40,41].

Consequently, while current findings suggest meaningful differences between adolescents with and without a mental health condition, further research using well-characterized clinical samples is needed to clarify the specificity and magnitude of these differences.

Data shows that social media experiences that pose a risk or represent a protective factor against suicidal ideation correspond to well-known offline risk and protective factors [41], which suggests that online interactions often mirror real-world social dynamics and their implicit challenges and support.

Several methodological challenges that might have contributed to the inconsistent findings in the literature to date are the heterogeneity of social media platforms, the variety of the measurements taken into account when assessing social media and mental health, clinical populations being underrepresented [34], data coming mostly from cross-sectional design studies that make it impossible to establish causality, reliance on self-reports that are susceptible to bias, not taking into account confounding variables (individual or environmental factors) [58], and the impossibility of conducting randomized control trials.

In line with the growing public and scientific concern, Jonathan Haidt argues in his recent book entitled The Anxious Generation that the increase in adolescent mental health disorders in the last twenty years may be linked to problematic social media use [59] (pp. 28–55). Although this perspective has attracted considerable attention, this claim does not constitute peer-reviewed empirical evidence and should be understood as an interpretation of existing research presented in a popular science book. At the moment, there is still no consensus on whether social media is causing the problem, or whether adolescents who have mental health vulnerabilities are more drawn to it, or whether there are other confounding variables that influence both social media use and mental health, or whether there is a reciprocal relationship with social media use and psychological symptoms where they reinforce each other [60,61].

While not neglecting the negative implications, it is important to acknowledge that adolescents also report important benefits from social media use such as self-expression, connection, reduction in social isolation, supportive communities, and easier access to mental health information [51,52,53,55]. However, gathering medical knowledge from social media platforms might be a double-edged sword that highlights the need to teach children and adolescents how to evaluate the quality of the information and the credentials of their sources.

Taking into consideration all of the above, we advocate for digital literacy training for both parents and youth [53], as well as for cultivating quality peer and family relationships that seem to contribute significantly to adolescents’ mental well-being [62]. However, while these public health strategies are valuable, the current evidence base remains insufficient to design highly targeted clinical interventions. Moving forward, the development of specific clinical protocols requires further validation within well-characterized clinical samples. Additionally, we emphasize the need for further longitudinal research that uses objective metrics and focuses on clinical populations of children and adolescents and how specific social media affordances interact with pre-existing vulnerabilities. Such data are essential for the development of targeted interventions for adolescents with mental health challenges and for the elaboration of evidence-based public health policies.

## 5. Limitations

Some limitations that must be taken into consideration when interpreting the findings of this review are the use of Scopus as the only database included in the search strategy, the heterogeneity of the included studies, the absence of a formal quality or risk of bias appraisal, which limits the ability to assess the reliability of the evidence, and its reliance on correlational and self-reported data that cannot establish causality. The narrative nature of this review may reduce reproducibility compared with systematic review methodologies. Consequently, the findings should be interpreted as a descriptive synthesis of the current literature rather than an exhaustive quantitative evaluation of the evidence.

The longitudinal studies included in this review varied considerably in their analytical approaches. Many employed multivariable regression models and adjusted for important confounders such as demographic characteristics, baseline mental health symptoms, and prior social media use. Nevertheless, the extent of covariate adjustment differed across studies, and residual confounding cannot be excluded. Therefore, although several studies reported modest and potentially bidirectional associations, these findings should be interpreted with appropriate caution.

The lack of universal definitions for key concepts (such as problematic social media use, active versus passive use) and the heterogeneity of the platforms make comparisons difficult. Although the studies included in the review were published after 2020, many of them do not include data collected in recent years, potentially overlooking emerging trends and newer features of social media platforms such as short form videos on TikTok. In order to strengthen the evidence base, further research would benefit from longitudinal cohorts of adolescents with diagnosed mental health disorders and designs including repeated measurements that allow for the examination of temporal and potentially bidirectional relationships between social media and mental health. To reduce recall bias and assess impact on well-being, questionnaires could be administered immediately after using various social media platforms. Additionally, objective metrics such as data logs and platform-derived usage metrics may provide a more accurate understanding of social media behaviors. Nevertheless, future research should investigate social media use motivation and outcomes for adolescents both with and without mental health disorders.

## Figures and Tables

**Table 1:** Main social media affordances and their mechanisms.

Mechanisms by Which Affordances Amplify Adolescents’ Mental Health Vulnerabilities	Affordances That Mediate Them
Cognitive (upward social comparison, disrupted self-concept development, social feedback, social inclusion and exclusion) [14]	Association; Personalization; Quantifiability; Synchronicity; Verifiability; Visibility [14].
Behavior (risky posting, a curated self-presentation) [14]	Anonymity; Bandwidth; Editability; Persistence; Replicability [14].
Neurobiological (an amplified stress reactivity and modification of reward processing) [14]	Availability; Variability of social rewards [14].

**Table 2:** Affordances and their proposed relevance to adolescent mental health.

Affordance	Proposed Mechanisms	Relevance to Adolescent Mental Health
Quantifiability (numerical social metrics such as likes and followers, unique to digital interactions; serves as an indicator of popularity and status [14])	Amplifies mechanisms such as upward social comparison, validation-seeking, and social exclusion, which are all associated with lower self-esteem and negative mental health outcomes [13,14]. Long-term reward processing is potentially affected by recurrent checking and reward-seeking, strategies used by adolescents to diminish the unpredictability of quantifiable social feedback (e.g., not knowing if and how many likes they will obtain) [14].	These mechanisms are particularly significant for adolescents’ mental health, as, compared to adults, adolescents from the general population show a more pronounced sensitivity to social feedbacks, with self-esteem registering higher fluctuations after digital social feedback [26,27]. Furthermore, an experimental study on subjects from the general population demonstrated that a reduction in likes has a stronger negative impact on adolescents than on adults [26].
Persistence (the period in which posts, comments, and photos remain accessible once shared [14])	High persistence is associated with a lack of freedom to experiment with identity, and increased fear of judgment and rumination about online events, therefore intensifying the need for a curated self-presentation and lowering well-being [14].	Moreover, although social platforms have expanded low-persistence tools in order to increase users’ comfort, adolescents report ongoing worry and vigilance, as screenshotting and saving of content remain possible [14].
Visibility, also termed publicness (the facility with which posts can be viewed and discovered by other people [14]; social media platforms allow their users to adjust audience size (public, semi-public and private), so visibility is usually an affordance that differs across users and that is associated with public self-perception [14])	Risky posting, curated self-presentation [14]. This variety of visibility encourages identity exploration among adolescents by trying different private or public selves [14].	A public, highly visible profile was associated in adolescents from the general population with approval anxiety and higher identity shifts than private self-presentation, concluding that high social exposure can influence how adolescents perceive themselves [14,28]. In contrast, manageable features of visibility (e.g., deleting, hiding) are being utilized by adolescents to reduce anxiety [28]. A recent study of around 2100 adolescents from the general population looked at the four main different activities by which adolescents navigate and express themselves on social media platforms (positive broadcasting, intimate broadcasting, intimate directed communication, and positive content consumption) and the outcomes visibility had on mental health [29]. Positive broadcasting, represented by sharing personal positive experiences, was associated with a more positive self-image, although quantifiability still adds some concerns about validation and social comparison. Intimate directed communication, characterized by private chats, exposes adolescents to peers’ opinions and accentuates social comparison, increasing anxiety and depression during identity development. Intimate broadcasting, defined as sharing publicly personal content, enhances approval anxiety, while social feedback or the lack of it triggers feelings of validation or rejection. The fourth feature, positive content consumption (e.g., scrolling through others’ profiles or posts), had the strongest association with upward social comparison and depression [29].

## Data Availability

No new data were created or analyzed in this study. Data sharing is not applicable to this article.
